# Standardization of cross-site biophysical studies of bovine insulin amyloids is challenged by structural polymorphism

**DOI:** 10.1007/s00249-025-01811-6

**Published:** 2026-01-21

**Authors:** Sofie Nyström, Davide Odino, Annalisa Relini, Claudio Canale, Raffaella Parlato, Yari Knelissen, Alessia Lasorsa, Wouter H. Roos, Patrick C.A. van der Wel, Søren V. Hoffmann, Nykola C. Jones, Vincent van Hemelryck, Jehan Waeytens, Vincent Raussens, Per Hammarström

**Affiliations:** 1 Department of Physics, Chemistry and Biology (IFM), Linköping University, Linköping, 581 83 Sweden; 2 Department of Physics, Genoa University, via Dodecaneso 33, Genoa, 16146 Italy; 3 Zernike Institute for Advanced Materials, University of Groningen, Groningen, The Netherlands; 4 Department of Physics and Astronomy, ISA, Aarhus University, Aarhus C, 8000 Denmark; 5 Spectralys Biotech, Université libre de Bruxelles, Bruxelles, Belgique; 6 Research in Drug Development – Pharmacognosy, Bioanalysis, Drug Discovery, Université libre de Bruxelles, Bruxelles, Belgique; 7 Structure et Fonction des Membranes Biologiques, Université libre de Bruxelles, Bruxelles, Belgique; 8 SciLifeLab, Linköping University, Linköping, Sweden

**Keywords:** Amyloid, Structural polymorphism

## Abstract

Protein structure can be analysed using a range of different techniques. Many of the available principal techniques for assessing protein structure are developed and optimized for natively folded proteins that are soluble. Amyloids are an alternative protein fold, often associated with protein malfunction and disease but are also found as the functional fold for some proteins. While functional amyloids, just like soluble globular proteins, commonly attain one specific and precise fold to exert its function, disease associated amyloids are often poorly soluble and structurally highly variable. This is known as structural polymorphism. To investigate to what extent molecular biophysics techniques can be standardized for studies of amyloid fibrils, five sites within the Horizon 2020 funded project MOlecular-Scale Biophysics Research Infrastructure (MOSBRI) addressed this issue via a joint research activity. For this study we selected bovine insulin as a convenient, accessible, and distributable amyloid model system. The benchmark comparable techniques for observing formed amyloid fibrils at the different sites were fluorescence spectroscopy of two amyloid ligands (ThT and pFTAA) and negative stain transmission electron microscopy. The outcome of this study yielded results with a large variability between different insulin amyloid fibril preparations, between sites, and within chemically identical preparations from one site. We mainly attribute these hard to control differences to the intrinsic polymorphic behaviour of insulin amyloid fibrils. We also present a range of experimental measurement techniques to highlight their potential use for studying amyloid structure and amyloid polymorphism.

## Introduction

Within structural biology and molecular biophysics there are attempts to reach standardized and benchmarked experimental methods and techniques. Structural polymorphism of disease associated amyloids exhibit a challenge when striving to design diagnostics, as well as therapeutic interventions, for the class of amyloid associated disease known as amyloidosis (Fandrich et al. 2018). The amyloid fold is an alternative conformation, rich in β-sheet structure. An amyloid fibril is comprised of many thousands of protein monomers that assemble through intermolecular interactions to form long strings of cross-β-sheets, with the β-strands aligned perpendicular to the fibril axis. While amyloids are mostly associated with disease in mammals, several other forms of life use amyloids to exert vital functions (Otzen and Riek 2019). Additionally, in concert with the developments in rational design of proteins and protein structure, the potential of the amyloid fold is being exploited as a future biomaterial (Otzen and Riek 2019; Pena-Diaz et al. 2024).

Protein biophysics and biophysical measurement techniques have evolved over many decades to detect and monitor both static and dynamic properties of natively folded proteins and protein complexes, leading to immense increase in the understanding of protein structure-function relationships. In turn, this has had large impact on understanding drug targets and development of therapeutics for diseases that are due to dysfunction of the native state of proteins. The inherent ability of many or most proteins to form amyloid fibrils has been known for several decades (Otzen and Riek 2019) and the number of proteins known to form amyloids associated with human disease is steadily increasing and was 42 different proteins in 2024 (Buxbaum et al. 2024). An increasing life-expectancy worldwide in combination with age being a dominating risk factor for many amyloid-associated diseases, is predictive of an increased number of patients afflicted by amyloid disease. However, while the native state of proteins largely follows the Anfinsen postulate that one sequence will result in one fold, albeit a dynamic or unstructured one, the amyloid fold formed from identical amino acid sequences can give rise to a large number of heterogenous structures. This variability is known as structural polymorphism. Such polymorphisms are a challenge for targeting amyloid structures in therapeutic interventions (Fandrich et al. 2018) but could also be utilized as a feature for targeting disease-associated amyloids in precision medicine while ignoring less aggressive amyloids. There is therefore both an increasing need and interest to understand the polymorphic nature of amyloid folds (Lutter et al.^2021^; Aubrey and Radford^2025^; Louros et al. 2023) in a manner that can allow the community of amyloid research to follow the successes of protein science and biophysics of the native state.

Increasing the knowledge of amyloid structure is of utmost importance regardless of the implementations of that knowledge. In the context of disease, it is vital to designate targets for diagnostics and therapeutics. The exploitation of amyloids as biomaterials is still only in an early stage. The possibilities given by Cryo-EM to obtain detailed high-resolution information of the static end state of amyloids found in disease has been paradigm shifting. However, this method is not optimal for following the dynamic changes as amyloid structure evolves over time. Although efforts towards this end are developing with Cryo-EM, Wilkinson et al. (2023); Lövestam et al. (2024) complementary analyses are necessary to follow these complex processes.

Structural polymorphism is a fundamental property of amyloid fibrils (Tycko 2015). This means that a single polypeptide or protein can adopt different types of amyloid fibril arrangements, based on even subtle differences in the environmental conditions. These amyloid polymorphs have distinct molecular structures, where the differences can be very dramatic. The differences in fibril structure can be associated with different biological properties, and different materials properties of the fibrils. Despite the functional importance of amyloid polymorphism, it is also a challenge to the amyloid research field. Studies of the same protein, performed by different research groups, can come to diverging conclusions, simply due to the formation of distinct polymorphs with different properties. This can hinder research processes in the field, leading to the demand for a better understanding of lab- or investigator-dependent differences in fibril polymorphism.

One key aspect of doing high-end biophysical measurements is to validate the quality and reproducibility of the measured sample. This is particularly relevant if several measurement techniques are utilized and several experimental sites and personnel are involved. We therefore set up a strategy for parallel amyloid formation at different sites using the same starting material in terms of protein and buffer.

**Fig. 1:**
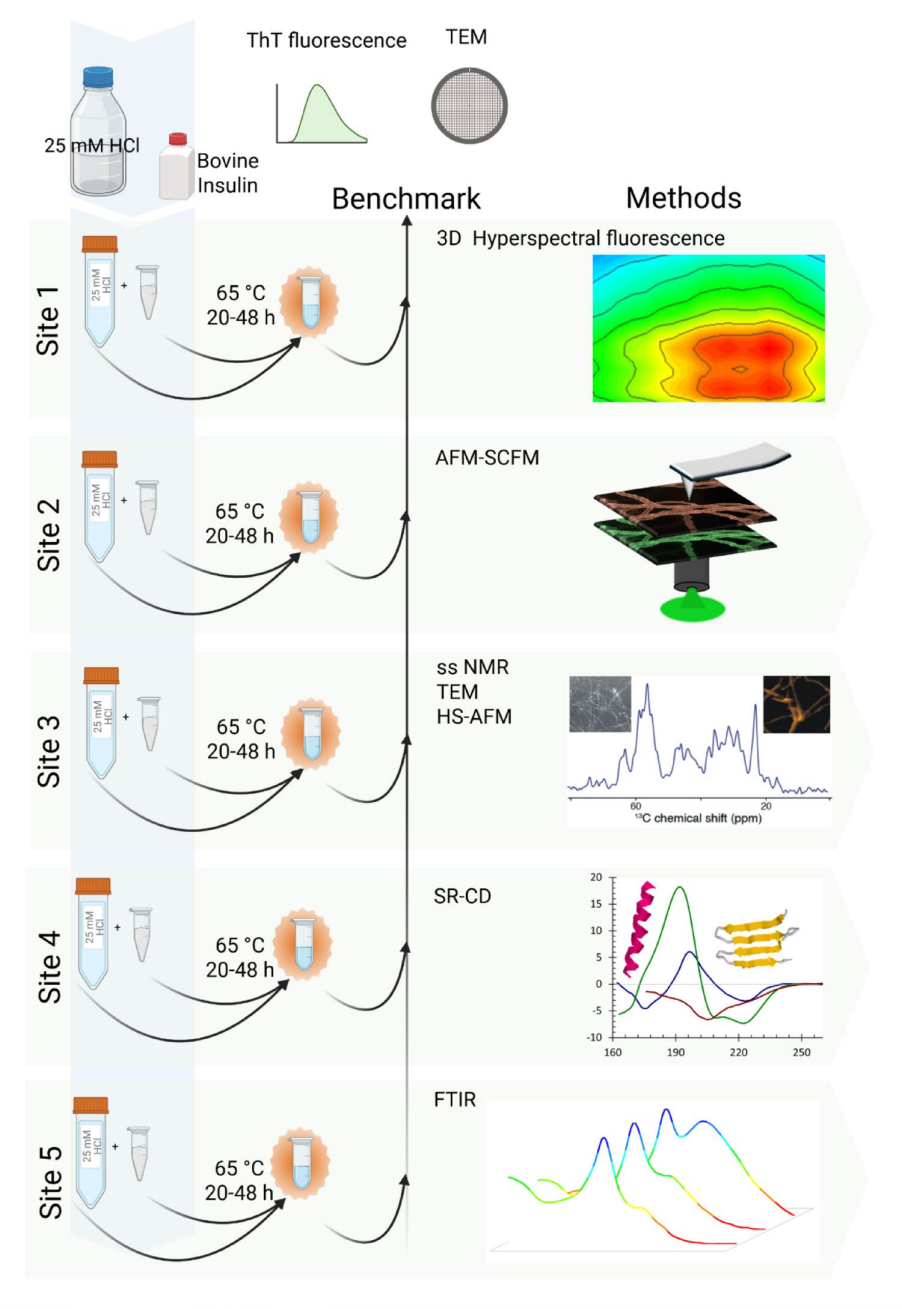
Schematic overview of the project. The same batch of lyophilized bovine insulin and solvent were distributed to five different sites within MOSBRI for fibril formation. Fibrils produced were sent to site 1 for benchmarking with ThT fluorescence and transmission electron microscopy (TEM), as well as LCO pFTAA hyperspectral fluorescence characterization. All sites used their respective fibrils to assess fibril characterization with different molecular biophysics techniques

Toward this aim, we herein undertook a multi-lab investigation of a widely used model system for amyloid formation (with relevance to biomedical contexts). We chose to study amyloid fibril formation by the bovine insulin polypeptide. Bovine insulin is commonly used for amyloid studies based on its similarity to human insulin, which is affected by biomedically important amyloid formation (Surmacz-Chwedoruk et al. 2016; Jimenez et al. 2002; Bouchard et al. 2000; Yuzu et al. 2020). This amyloidogenic peptide is known to undergo amyloid formation, under quite simple experimental conditions, making it an ideal target for our study. We distributed the same peptide to five different sites for a joint research activity (JRA) within MOSBRI (Fig. 1). With the use of the basic benchmarking methods thioflavin T (ThT) fluorescence detection and transmission electron microscopy (TEM), as well as hyperspectral characterization of LCO stained fibrils, it was found that there were substantial variations of the formed amyloid fibril preparations from seemingly identical samples, likely attributed to fibril polymorphism. In this paper we also demonstrate the applicability of a range of different biophysical techniques that can be utilized to explore the structure of amyloid fibrils, using bovine insulin as a model protein.

## Methods

Lyophilized insulin purified from bovine pancreas was purchased from Sigma-Aldrich (Cat#6634) and 10–20 mg dissolved in 25 mM HCl in milliQ water (pH 1.6) at site 1. The protein was filtered through a 0.22 μm PVDF filter using a syringe before protein concentration determination by absorbance measurements using A_277_ and ε = 5840 M^− 1^cm^− 1^. The protein was diluted in 25 mM HCl in milliQ water to a stock concentration of 320 µM (on a monomer basis) and distributed to the 5 different sites. Separate containers of lyophilized insulin powder (20–25 mg) and solutions of 25 mM HCl for setting up fibrillation reactions at each site and 0.22 μm PVDF filters were also distributed. Fibrils from site 1 were formed by incubation of 320 µM insulin for 65 °C (heated water bath) for 24 h. Fibril preparation “Site 1A” were distributed to all other sites.

At each site, the reconstituted native protein shipped by site 1 was diluted to a final concentration of 320 µM in 25 mM HCl and subjected to heating at 65 °C for 20–24 h. Amyloid content was deduced by ThT fluorescence measurement at each site by 10–25 fold dilution of aliquots of fibril sample with PBS buffer (140 mM NaCl, 27 mM KCl, 10 mM PO_4_^3−^, pH 7.4) containing 20 µM ThT. Fluorescence was measured by excitation at 440 nm and emission monitored at 470–650 nm using the equipment available at each site. Sites 1, 4 and 5 found poor ThT signal after 20 h incubation, so the incubation was prolonged for another 18–24 h whereafter all samples displayed clear ThT fluorescence. Each of the sites 2–5 proceeded with their self-obtained insulin amyloid sample, prepared using the lyophilised sample and buffer sent by site 1 and used for their specific measurements and using the standard equipment and materials available at each site. Finally, aliquots of these insulin amyloids from each site were shipped to site 1 for comparative analysis of amyloid structure by TEM and fluorescence spectroscopy using amyloid fluorescent ligands.

### Thioflavin T intensity fluorescence measurements

**Scheme 1:**
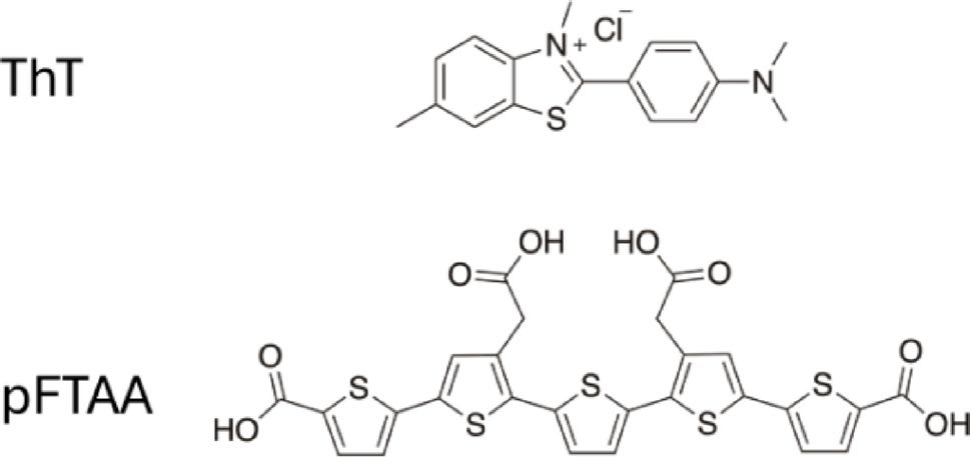
Chemical structures of Thioflavin T (ThT) and the luminescent conjugated oligothiophene (LCO) pentameric formyl thiophene acetic acid (pFTAA)

An increase in Thioflavin T (ThT) fluorescence intensity is one of the major hallmarks of amyloid presence in solution samples (Gade Malmos et al. 2017; Naiki et al. 1989; Xue et al. 2017). When the ThT molecule (scheme 1) is free in solution it can freely rotate around the central C-C-bond between the benzothiazole and the anilino moiety, resulting in self-quenching (Lindgren et al. 2005; Srivastava et al. 2010). When the molecule binds to an amyloid fibril, this limits the rotational freedom and its fluorescent property is restored when the molecule is excited at 440 nm. While ThT that is free in solution can give rise to weak fluorescence emission peaking above 500 nm, binding to amyloids typically gives rise to a dramatic increase in fluorescence emission intensity with a single peak around 476–485 nm.

ThT intensity measurement is very suitable as a first screen for amyloids in an unknown sample in solution. It can also be used in kinetic experiments to deduce the time it takes for amyloids to form *in vitro*. It is worth noting that ThT fluorescence is sensitive to pH and signal can drop drastically at low pH (Lindgren et al. 2005; Hackl et al. 2015).

At site 1, ThT fluorescence was measured concurrently on all samples received from all sites. One sample aliquot from each site was heavily vortexed, 10 µl of each sample in technical triplicates were aliquoted to 90 µl of ThT assay buffer (140 mM NaCl, 27 mM KCl, 10 mM PO_4_^3−^, pH 7.4, 11 µM ThT) in a 96-well plate (Corning costar 3880, non-treated black with transparent bottom, half area wells) to reach a final concentration of 32 µM insulin (monomer equivalent) and 10 µM ThT. The plate was measured bottom up in a Tecan infinite M1000 pro plate reader (Tecan, Austria), with excitation set to 440 nm and emission spectra were collected between 450 and 650 nm.

### Negative stain transmission electron microscopy

Negative staining applies a heavy metal stain to the deposited specimen in a carbon coated holey copper grid, which renders a dark background around the fibril sample providing contrast under the electron beam. Site 1 performed negative stain transmission electron microscopy (TEM) of four different sample preparations from site 1 and the four different sample preparations from sites 2–5. 5 µl samples were applied on carbon-coated 400-mesh copper TEM grids (Carbon-B, Ted Pella inc.) and left to adsorb for 2 min. Excess sample was removed with filter paper by gently touching the edge of the TEM grid. 5 µl of milli-Q water was applied to the TEM grid for washing and blotted dry with filter paper. 5 µl of 2% w/w uranyl acetate (in milli-Q water) was applied to the TEM grid and incubated for 30 s. Each grid was carefully blotted dry as before and left to dry at room temperature. Images were obtained using a Jeol JEM1400 flash transmission electron microscope operating at 80 kV equipped with a Gatan digital camera.

Site 3 also performed negative stain TEM, both on fibrils prepared at site 1 and on fibrils made at site 3 according to the shared protocols. Thus, insulin fibrils were prepared at 320 µM in HCl buffer (25 mM). The on-site prepared fibrils were allowed to aggregate for 20 h at 65 °C. The fibrils were suspended in Milli-Q water, at a concentration of ~ 32 µM (monomer equivalent). The fibrils were applied to plain carbon-coated 200-mesh copper TEM grids (SKU FCF200-Cu-50, Electron Microscopy Sciences, Hatfield, PA), which had been glow-discharged before use. A drop of sample was put on the grids for 1 min, the excess was removed by blotting, and then the grids were washed using Milli-Q water. As a negative stain agent, uranyl 2% (wt/vol) acetate was used, which was left on the grid for 1 min and then removed by blotting. Finally, the grids were left to air-dry prior to imaging. Imaging was carried out on a Philips CM120 electron microscope at 120 kV, with images captured using a Gatan slow-scan CCD camera.

### Analysis using the amyloid conformation sensitive fluorescent dye pFTAA

Amyloid conformations can be discriminated using fluorescence spectroscopy and microscopy of samples stained with conformation sensitive dyes (Bjork et al. 2023), with such dyes being used both for measuring samples in solution and for microscopy (Nystrom et al. 2017). The method can be used with a broad range of fluorescent molecules with different excitation and emission wavelengths and most importantly, with differential binding propensity to various amyloid structures, making them versatile tools for screening and differentiation of amyloid polymorphs (Nystrom et al. 2013).

From previous studies it is known that structural differences of amyloids can be observed by pFTAA fluorescence spectra of the commercial drug insulin, both *in vitro* and from *in vivo* patient biopsies (Yuzu et al. 2020). Luminescent conjugated oligothiophene (LCO) pFTAA (Aslund et al. 2009) (Scheme 1) was therefore utilized here. An assay solution of 300 nM pFTAA in PBS buffer (Medicago, 140 mM NaCl, 27 mM KCl, 10 mM PO_4_^3−^, pH 7.4) was prepared. 90 µl of the assay buffer was added to wells in a Costar 3880 plate, to which 10 µl of the insulin amyloid samples from each site were added in technical triplicates. In addition, triplicates of 10 µl of native insulin and 10 µl of 25 mM HCl in assay buffer were added as references.

Hyperspectral 3D excitation and emission scans were measured using a Tecan infinite M1000 pro plate reader with the following settings: excitation at every 5th nm between 380 nm and 480 nm and emission at every 5th nm between 500 nm and 700 nm. Slits for excitation and emission were set to 5/5 nm and gain was set to 120.

### Correlative AFM-confocal microscopy

Site 2 performed correlative Atomic Force Microscopy (AFM) and confocal microscopy. Although the differential binding propensity of conformation-sensitive dyes to various amyloid structures makes them a valuable tool for the characterization of amyloid polymorphs (Nystrom et al. 2013), part of the aggregation product can be undetectable via fluorescence microscopy and spectroscopy due to its poor or total lack of affinity with the dye molecules. Recently, it was demonstrated that the presence of protein monomers labelled with fluorophores changes the protein aggregation propensity (Cosentino et al. 2019; Jadavi et al. 2023). In this scenario, the use of a correlative technique coupling fluorescence microscopy with a high-resolution label-free microscopy, i.e., the atomic force microscope, can be very informative, allowing a complete characterization of the aggregation process. Fibrils prepared in site 1 and site 2 were studied and compared. Samples were labeled with the LCO pFTAA using the same method and dye concentration detailed earlier (300 nM). In this work, it was not possible to apply super-resolution microscopy, as proposed in the past for insulin fibrils, Cosentino et al. (2019) since the dye does not allow this modality for different reasons. First, the fluorophore absorption and emission are not suitable for STED in our system that has a laser depletion beam at 775 nm. Second, pTFAA and other conformation sensitive dyes do not fit the requirements for a good STED fluorophore in our setup (Sednev et al. 2015; Blom et al. 2017). Therefore, we employed scanning confocal fluorescence microscopy (SCFM) in conjunction with AFM. To achieve reliable results, we prepared diluted fibril samples, minimizing the overlap between adjacent fibrils in confocal images, which would otherwise hinder correlation with AFM.

Insulin fibrils for fluorescence inspection were deposited onto glass coverslips. Prior to deposition, the substrates were treated with oxygen plasma using a Tucano system (Gambetti Kenologia, Italy). The coverslips underwent three irradiation cycles of 180 s each at 100 W with an oxygen flow of 13 SCCM. Aliquots of the aggregated insulin solution were diluted 100-fold (resulting in 0.02 mg/ml) in Milli-Q water, and a 50 µl drop was deposited on the coverslips immediately after the plasma treatment. After a one-minute incubation, the samples were thoroughly rinsed with Milli-Q water to remove salts and unbound material.

Correlative AFM-SCFM images were acquired using a NanoWizard IV AFM head (Bruker, USA) mounted on a Stellaris-8 STED microscope (Leica Microsystems, Germany). AFM images were collected in PBS using tapping mode with triangular silicon nitride cantilevers (NPG, Bruker, MA, USA) that have a tip curvature radius between 20 and 60 nm and a nominal spring constant of 0.24 N/m. Confocal images were recorded using a HyD X2 detector in the 651–750 nm range. An HC PL APO CS2 100X/1.4 NA oil-immersion objective (Leica Microsystems, Mannheim, Germany) was used with a scan rate of 200 Hz for 1024 pixels per line and an accumulation of 4 lines. The conventional microscope stage was replaced by an AFM-compatible stage to minimize mechanical noise coupling to the cantilever. Using the Direct Overlay module of the AFM software, the optical images from the confocal microscope were overlaid onto the AFM images. Sequences of cantilever images were acquired in reflection mode with the confocal microscope at nine predefined positions within a 33 μm × 33 μm calibration area. To avoid photobleaching, laser excitation was set to 647 nm, far from the fluorophores’ excitation wavelengths, using less than 1% of the maximum laser power. The cantilever images were captured with a HyD S1 detector (470–500 nm), at a scan rate of 400 Hz for 512 pixels per line, with an average over 16 lines. The ratio of co-localization is provided by the Mander’s coefficient (M1), which quantifies the fraction of SCFM image pixels corresponding to fibrils in both SCFM and AFM images, normalized by the total number of pixels associated with fibrils in the AFM image (Jadavi et al. 2023). M1 was calculated with custom made MATLAB software. In particular, we identified fibrillar aggregates by applying a threshold on the pixel intensity (representing the height in AFM and the fluorescence intensity in SCFM images) based on the background noise level. We obtained binary images where the pixels corresponding to fibrils (intensity above the threshold) are assigned the value 1 and the background pixels are assigned the value 0. The Mander’s coefficient was obtained using the following equation:

1 $$\:{M}_{1}=\frac{\sum_{i}{AFM}_{i,\:coloc}}{\sum_{i}{AFM}_{i}}$$

Where $\:{AFM}_{i,\:coloc}$ is a pixel where a fibril is present in both AFM and SCFM images. $\:{AFM}_{i}$ is a pixel where the fibril is present in the AFM image.

### High-speed atomic force microscopy (HS-AFM)

Site 3 performed high-speed atomic force microscopy (HS-AFM) measurements of fibrils prepared at site 1 and site 3. While high-speed measurements are not essential to characterise the fibrils, HS-AFM generally provides a similar - and sometimes higher - resolution compared to a common conventional AFM setup, among others due to the miniaturised tip and cantilever (Ando 2018).The measurements were done in amplitude modulation tapping mode using a HS-AFM (RIBM, Japan). A cantilever (USC-F1.2-k0.15, NanoWorld, Switzerland) with nominal spring constant of 0.15 N/m was used in the measurements. Measurements of both groups of fibrils were performed under similar conditions. A 2 µl droplet of the 320 µM fibril solution was incubated on mica for approximately 20 min. The sample was then washed with 10 µl of PBS before it was placed in a PBS (pH 7.4) environment in the AFM chamber where measurements took place. All AFM measurements were done at room temperature in liquid.

### Solid-state NMR analysis

Unlabeled insulin fibrils were prepared as described above (Site 3). Fibrils were recovered from the aggregation solution by centrifugation and packed into a solid-state NMR sample holder (3.2 mm MAS rotor from Bruker Biospin). 15 mg of unlabeled insulin fibrils aggregated for 20 h at 65 °C were loaded into 3.2 mm zirconia MAS rotors (Bruker Biospin, Billerica, MA) by pelleting directly into the rotor using an ultracentrifugal packing tool. This was done at approximately 130,000 × g in a Beckman Coulter Optima LE-80 K ultracentrifuge equipped with an SW-32 Ti rotor (Mandal et al. 2017). Prior to sealing the rotor, excess water was removed, and a spacer insert was placed between the sample and the drive cap. The ssNMR sample was measured on a Bruker Avance NEO 600 MHz spectrometer equipped with a 3.2 mm MAS probe with an HCN Efree coil (Bruker Biospin). Solid-state NMR experiments were conducted on the hydrated, unfrozen samples using magic angle spinning (MAS) at 12 kHz and 278 K. One-dimensional ^13^C spectra were recorded with the following parameters: a ^1^H 90° pulse duration of 2.5 µs (~ 100 kHz rf power) and a ^13^C 90° pulse of 5 µs (~ 50 kHz) for cross polarization (CP) and direct polarization (DE). During acquisition, ^1^H decoupling was performed using the TPPM scheme with a pulse length of 5.8 µs and an RF field strength of ~ 83 kHz. Chemical shifts for ^13^C ssNMR spectra were referenced indirectly using adamantane as a secondary standard, relative to aqueous DSS. Morcombe and Zilm (2003) Spectral processing was carried out using NMRPipe, Delaglio et al. (1995) and visualizations were generated in CcpNmr Analysis v2.5 (Stevens et al. 2011).

### Synchrotron radiation CD

Through the measurement of the differential absorbance of left and right circularly polarised light, circular dichroism spectroscopy (CD) is used to study chiral molecules. This commonly used biophysical technique involves the measurement of spectra over a range of wavelengths yielding broad structural information. For proteins in particular, CD spectra recorded in the far-UV (< 240 nm) reflect the secondary structure of the molecule, where the CD signal arises from the differing angles between the amino acid residues defining the various types of structures, such as alpha-helices, beta-sheets and turns. With distinct spectral forms for these structures, CD spectroscopy can be used to estimate the proportions of secondary structure elements in a protein and to follow changes in the conformation when subjected to e.g. a change in temperature or in the sample conditions (buffer, pH etc.). Measurements are quick, require no staining or complex sample preparation and are typically carried out in solution using only a small amount of sample down to a few tens of µL.

At site 4, synchrotron radiation CD (SR-CD) is measured using the AU-CD beam line of the ASTRID2 synchrotron light source (ISA, Department of Physics and Astronomy, Aarhus University) (Miles et al. 2007, 2008). While benchtop CD spectrometers are able to measure from the visible into the far-UV, the light sources used limit their ability to measure lower than 190 nm. Using synchrotron light as a source, the intensity of the available light continues to increase below 190 nm and remains high, allowing measurement further down in wavelength and is ultimately limited only by the absorbance of water and/or buffers. This feature is crucial for being able to accurately and fully measure bands below 200 nm for reliably determining secondary structure.

The CD spectrum of each sample was measured at 25 °C from 330 to 170 nm in 1 nm steps with a dwell time of 2 s/pt. Sample spectra were measured at least in triplicate and averaged, with a reference baseline of the 25 mM HCl buffer, measured in triplicate, subtracted. Measurement of CD spectra were carried out using a nominal 0.1 mm pathlength cell, determined accurately to within 0.1 micron using an interference method (Hoffmann et al. 2016). At low wavelengths, there is significant absorbance from the sample and buffer, so all data are cutoff at a wavelength defined by the high voltage being applied to the detector, at a level which indicates that the light intensity is too low to accurately measure a CD signal; this ranged from 183 to 185 nm for these samples, depending on the concentration measured, but is otherwise limited by the concentration of highly absorbing Cl^−^ ions. The correct operation of the CD spectrometer was confirmed through the measurement of (S)-(+)-camphor-10-sulphonic acid (CSA) (Miles et al. 2003). 

The concentration of the sample was confirmed by the absorbance at 205 nm, Anthis and Clore (2013) which was recorded simultaneously with the CD. Analysis using SSCalcPy (Hoffmann et al. 2025) with the CDSSTRPy method and the AU-SMP180 reference set.

### Infrared spectroscopy measurements

Attenuated total reflection Fourier- transform infrared (ATR-FTIR) spectra were recorded at site 5 using a Bruker Equinox55 infrared spectrophotometer equipped with a LN_2_-MCT detector at a resolution of 4 cm^− 1^. The spectrometer was continuously purged with dry air. 0.5 µL of the sample was deposited on the Specac diamond ATR, dried with a nitrogen flow and 128 scans were averaged for sample and background. The spectra were normalized on the area of the amide I and amide II bands (from 1760 to 1478 cm^− 1^).

## Results

### Insulin amyloid fibril formation demonstrates polymorphism

While monomorphic preparations of insulin amyloid fibrils have been reported (Suladze et al. 2024), it is established that insulin amyloid fibrils are structurally polymorphic (Jimenez et al. 2002; Zhou et al. 2024). Insulin amyloid polymorphism is dependent on fibril formation conditions (Psonka-Antonczyk et al. 2012; Ziaunys et al. 2024). Amyloid fibril polymorphism is a common feature of amyloid formation but is a hurdle for standardization of protocols and for the comparison between experiments performed at different institutes.

### Benchmarking activities

#### ThT fluorescence intensity and negative stain TEM

**Fig. 2:**
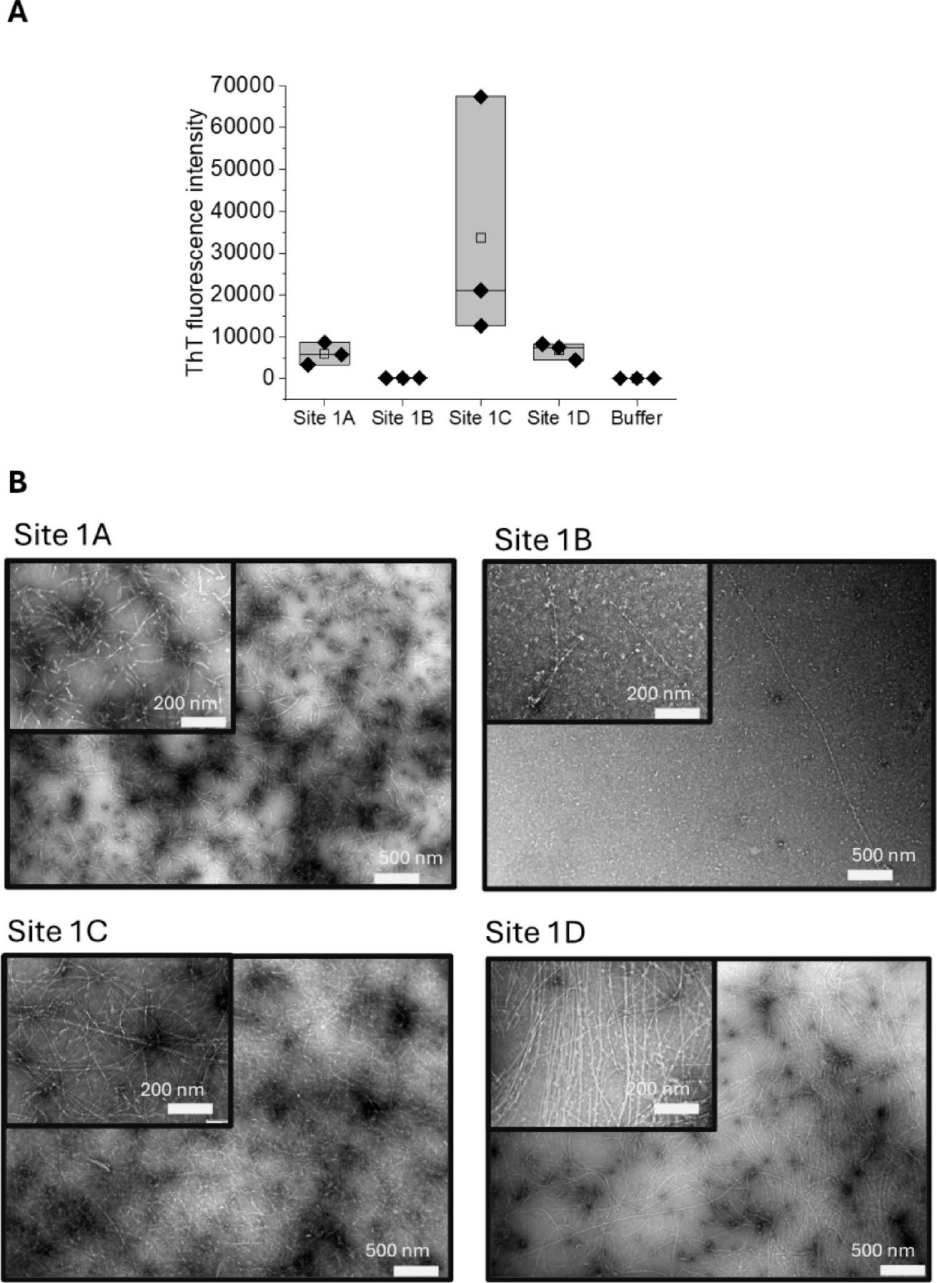
**A** ThT fluorescence intensities of various insulin fibril samples from site 1 assayed with the same assay solution and fluorescence plate reader. **B** Negative stain TEM micrographs of insulin fibrils from site 1. All four samples were prepared by the same person using the same protein, protocol and equipment but at different occasions over the course of five months. Samples 1C and 1D were prepared at the same time, side-by-side, from the same stock solution and in the same buffer

Due to the known conditional dependence of insulin amyloid polymorphism, we set out to produce insulin amyloid fibrils under identical chemical conditions, that is, using the same protein and buffer stocks but prepared at different sites. To this end, insulin purified from bovine pancreas was purchased as lyophilized powder, distributed to the various sites for solubilization to be subjected to amyloid forming conditions as described in the Methods section. Fibril preparation 1 A was distributed to all sites for further analysis. At site 1, the procedure was repeated several times with a few months’ interval, by the same person using the same protein and equipment, producing samples 1A to 1D. Site 1C and site 1D samples were run side-by-side from the same stock solution at the same time, yet the amyloid content and ultrastructure displayed large variability, as determined by ThT fluorescence intensity (Fig. 2A) and negative stain transmission electron microscopy (TEM) (Fig. 2B). The ThT intensity of the technical replicates of the site 1 A sample reached an intensity of 3300 to 8800 AU. TEM micrographs showed that the sample contained large amounts of short fibrils that appeared to be multi-filamentous, with a few intermediately long single filamentous fibrils also detected. The ThT intensity of the site 1B sample barely exceeded that of free ThT in PBS buffer, 140–220 AU for sample 1B compared to 100 AU for ThT in buffer (Fig. 2A). In agreement with the poor ThT signal, the TEM results confirmed low fibril content in the sample, with very few but long single filamentous fibrils found deposited on the grid (Fig. 2B). In addition, small spherical aggregates were found distributed on the grid surface, as well as being associated along the fibril surface, that may be prefibrillar aggregates with low response to the ThT assay. Site 1C and 1D samples were run side-by-side under identical conditions with the same starting material at the same time. Still, for the site 1C sample the variability of ThT intensity for technical replicate was large, spanning between 12,000 and 67,000 AU, while the site 1D sample displayed both lower and less varied ThT signal between the technical replicates (4300–7800 AU) (Fig. 2A). TEM images confirmed inter-sample variability (Fig. 2B). The 1C sample contained long, single filamentous and curved fibrils that sometimes clustered into large, densely packed bundles. TEM analysis of the site 1D sample showed that it contained both the curved fibril type of site 1C and a substantial amount of straight multi-filamentous fibrils that appeared to be decorated with spherical structures all along the fibril. Such spheres were also present, but were less abundant, between the fibril bundles, indicating that the spheres are attached to the fibrils (Fig. 2B). Note that site 1C and 1D samples did not display a significant increase in ThT fluorescence after 24 h incubation, so the incubation was prolonged for an additional 24 h for these two samples.

**Fig. 3:**
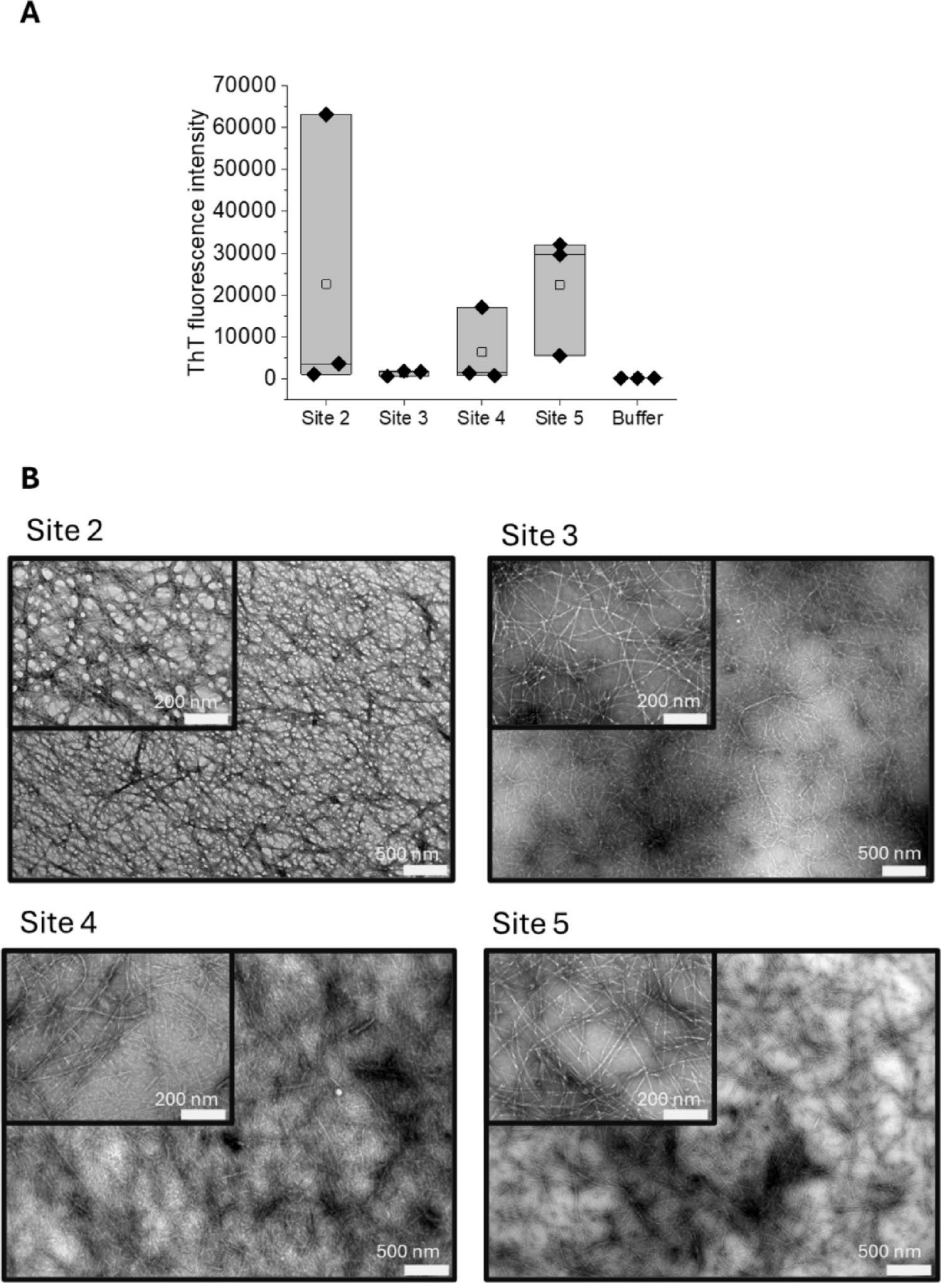
**A** ThT fluorescence intensities of various insulin fibril samples from sites 2–5 assayed with the same assay solution and fluorescence plate reader. **B** Negative stain TEM micrographs of insulin fibrils from sites 2–5

Reconstituted insulin from the same protein batch as site 1C and 1D samples above, together with buffer for amyloid formation was distributed to different sites for independent amyloid fibril formation according to the same protocol as above (Fig. 1). Samples of the generated fibrils at the different sites were then sent back to site 1 for analysis by ThT fluorescence (Fig. 3A) and TEM (Fig. 3B) for comparison side by side. Note that also site 4 and site 5 samples reached ThT signal increase corroborating amyloid formation only after an additional 18–24 h of incubation.

Again, recording of fluorescence intensity from technical replicates resulted in large fluctuations in ThT intensity within each sample as well as between samples from different sites. The site 2 sample exhibited ThT fluorescence ranging between 1000 and 63,000 AU (Fig. 3A). TEM analysis of this sample revealed densely packed fibril webs covering the grid (Fig. 3B). The ThT intensity from the site 3 sample was lower and less variable, 500–1800 AU (Fig. 3A). Here, almost exclusively mono-filamentous, long and straight fibrils were detected on the grid (Fig. 3B). The distribution of ThT intensities between replicates for the site 4 sample was large, spanning 700 to 17,000 AU (Fig. 3A). The electron micrograph showed fragmented, often multi-filamentous fibrils in combination with spherical structures (Fig. 3B). Finally, the site 5 sample showed an intermediately high ThT intensity spanning from 3800 to 28,000 AU (Fig. 3A). When analysed by TEM, homogenous, straight multi-filamentous, ribbon-like fibrils were observed (Fig. 3B). While all samples were analyzed at the same time with identical settings at site 1 there was a variation in the time gap between fibril formation at the different sites and these measurements. Fibrils were stored at room temperature in the interim period.

#### Hyperspectral fluorescence 3D-scans using the amyloid conformation sensitive fluorescent probe pFTAA

Amyloid conformation sensitive dyes have been developed and have mainly been used for amyloids from proteins associated with neurodegenerative disease (Bjork et al. 2023). They have also been utilized for detection and discrimination between amyloid subtypes of systemic amyloidosis (Westermark et al. 2025) and iatrogenic insulin amyloid *in vivo* (Yuzu et al. 2020). Previous data demonstrated that bovine insulin can generate distinct amyloid structures in the absence or presence of a reducing agent and we have shown that this polymorphic difference can be detected by using conformation sensitive fluorescent amyloid dyes (Psonka-Antonczyk et al. 2012). Hence similarities and differences of fibrils (i.e. fibril polymorphism) should be detected using this rather straight forward approach.

Here we used an LCO, pFTAA, to detect differences in amyloid structure of the insulin fibril preparations from different sites. The dye was mixed with the different insulin amyloid samples in a 96 well plate and hyperspectral 3D scans fluorescence assessment allowed analysis of fluorescence emission between 500 and 700 nm as result of excitation between 380 and 480 nm. Just as for the ThT measurements, the fluorescence intensity tended to vary between the technical replicates, but the spectral shift signatures remained consistent within each sample. The triplicate with highest maximum intensity for each sample type was selected for further evaluation below.

**Fig. 4:**
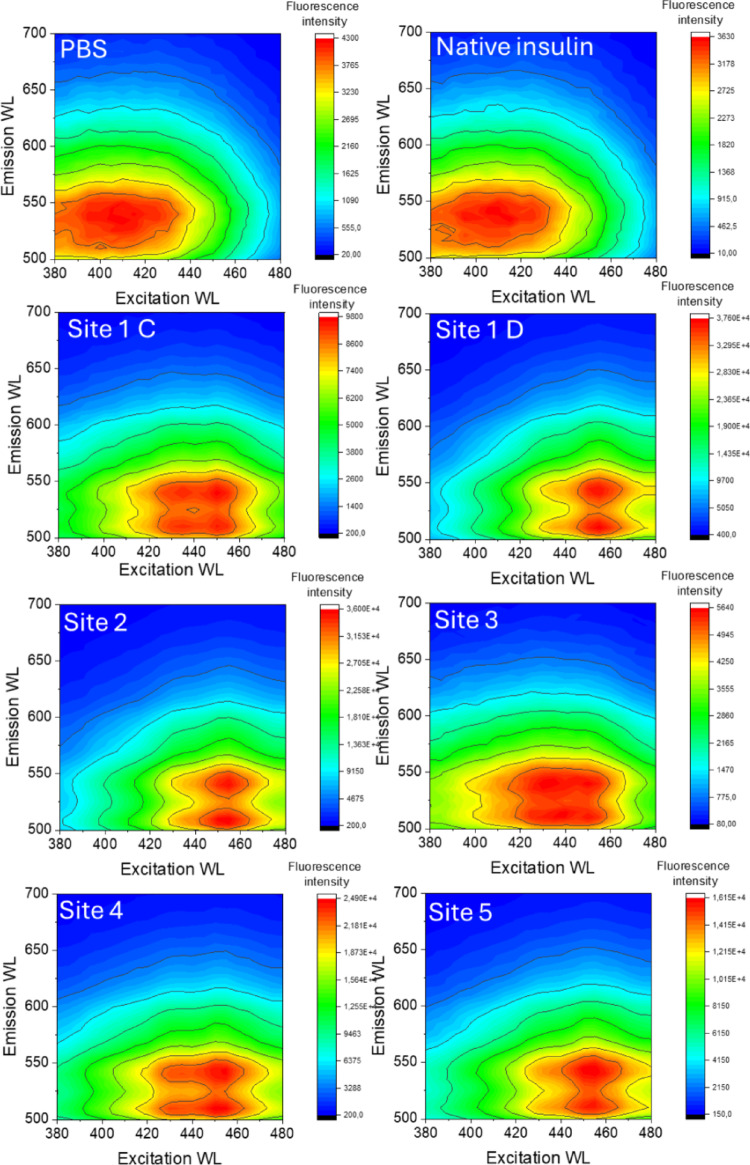
Hyperspectral 3D scans of pFTAA samples. The images display a matrix of excitation and emission fluorescence with intensities illustrated as various heat map colours. Samples from free pFTAA in PBS buffer, native insulin, and various insulin fibril samples from all sites were assayed with the same pFTAA assay solution and fluorescence plate reader

**Fig. 5:**
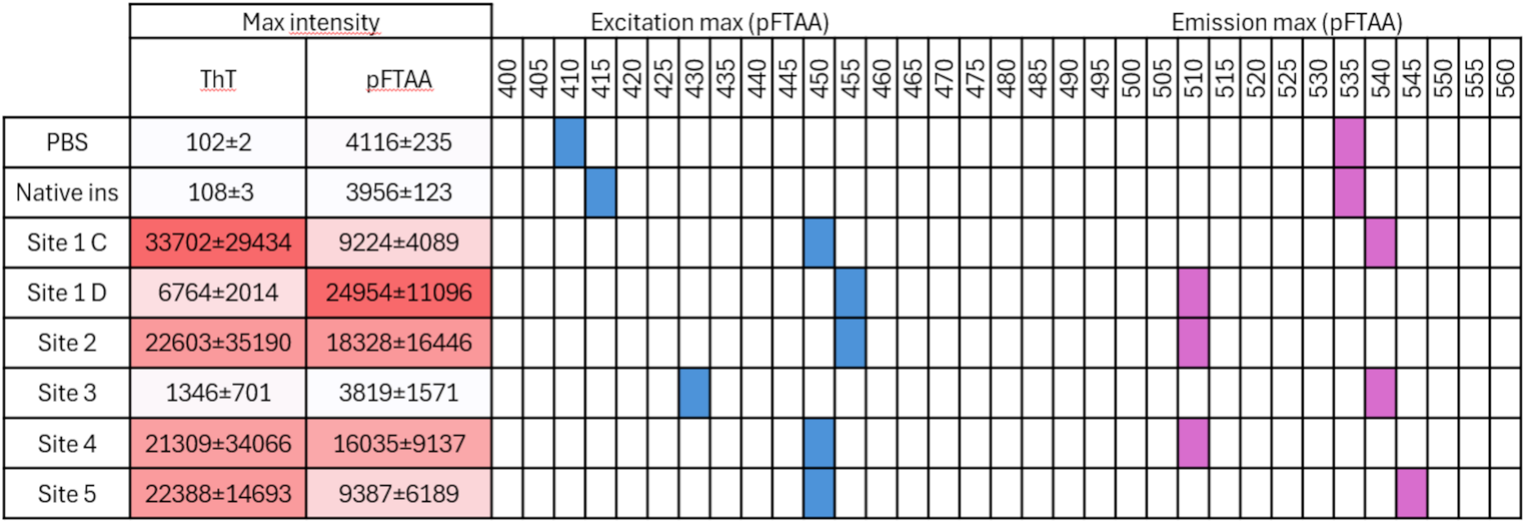
Summary of fluorescence data from ThT and hyperspectral analysis of pFTAA fluorescence. The chart summarizes the variation of fluorescence intensities and excitation and emission fluorescence maxima for the different samples. Samples from free ThT or pFTAA in PBS buffer, native insulin, and various insulin fibril samples from all sites were assayed with the same assay solution for the respective fluorescence probe and fluorescence plate reader

Firstly, the data from pFTAA demonstrated a clear red shift in excitation when pFTAA binds to the insulin amyloid as compared to the free probe by itself in PBS or together with native insulin (Fig. 4). The pFTAA spectra bound to fibrils reverts from a featureless broad emission maximum to show a double emission peak feature at around 510 nm and 540 nm, while with varying intensities between the two peaks (Nilsson et al. 2018). These spectral shifts are consistent with previous studies of pFTAA binding to insulin amyloid fibrils (Yuzu et al. 2020; Psonka-Antonczyk et al. 2012) and other amyloid fibrils e.g. Aβ1–42 (Nilsson et al. 2018). Secondly, however, scrutinizing the hyperspectral data of each sample the spectral profiles varied widely between the samples (Fig. 4). Most similar were the hyperspectral profiles from pFTAA bound to site 1D and site 2 samples (Figs. 4 and 5). Overall, these data corroborated with the ThT and TEM results that the different insulin amyloid samples were highly polymorphic.

### Additional biophysical methods to characterize amyloid fibrils

Each site utilized their specific measurement techniques on their independently produced fibrils and made some comparisons with fibrils made at site 1.

#### Correlative AFM-SCFM

**Fig. 6:**
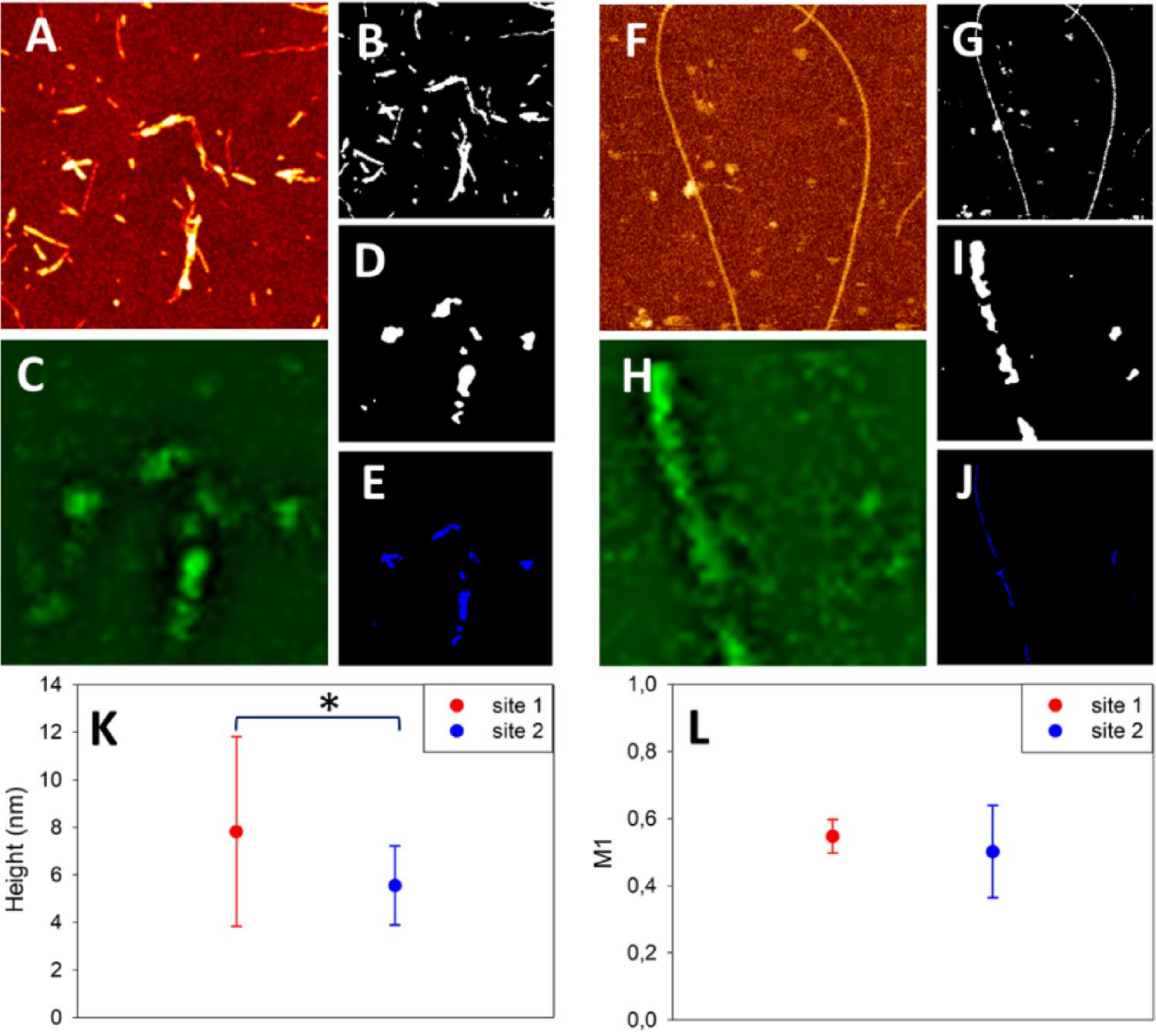
Representative AFM images of site 1 (**A**) and site 2 (**F**) fibrils. The scan size of both images is 3.5 μm x 3.5 μm. Corresponding SCFM images are shown in **C** and **H**. Binary images (**B**,** D**,**G**,** I**) obtained by thresholding the AFM (**B**,** D**) and SCFM (**G**,** I**) images to isolate the fibrillar aggregates from the background noise. AFM trace overlaid with the pFTAA fluorescence image (**E**,** J**). The fibril thickness is shown in **K** (mean and standard deviation). Amyloid aggregates from site 2 are longer and with a well-defined thickness. Fibrils from site 1 are thicker and present a broader distribution (*=*P* < 0.05). The Mander’s coefficient M1(Eq. 1) for the fibril populations of both sites is reported in **L**

Fibrillar insulin was observed after deposition on a glass coverslip using AFM and SCFM. From AFM analysis we detected the presence of fibrils in both of the samples prepared at sites 1 and 2. The morphology of the fibrils was however not the same. Fibrils from site 2 were generally longer, while fibrils from site 1 were shorter and tended to associate both laterally and in supercoiled structures (Fig. 6A-E). As a result of this, site 1 fibrils displayed a broader height distribution and a higher average thickness (Fig. 6K). Specifically, the mean height of site 1 fibrils was 7.8 ± 4.0 nm, whereas the mean height of site 2 fibrils was 5.5 ± 1.7 nm. In both cases we were able to recognize the same aggregates in AFM and SCFM images, but upon closer inspection the number of aggregates displayed in the AFM images was higher in comparison with confocal images. The Mander’s coefficient M1 (Eq. 1) calculated for site 1 fibrils was 0.55 ± 0.05, while for site 2 fibrils it was 0.50 ± 0.14. Although the ratio of labelled fibrils was comparable between the two sites, the measurements for site 1 fibrils showed significantly less variability. Similar results were found in the *in vitro* aggregation of insulin and other protein/peptide solutions in the presence of a fraction of labelled monomers (Jadavi et al. 2023; Cosentino et al. 2019).

In these previous cases we concluded that, in a scenario dominated by polymorphism, labelled monomers could follow only selected aggregation pathways. With the present work, we broaden this perspective, showing that also LCO based conformation-sensitive probes exhibit selectivity, effectively tagging only a subset of amyloid aggregates, at least at the dye concentration employed in this work (300 nM). It is notable that even within a single long fibril only one half of the fibril visible with AFM was stained with pFTAA (Fig. 6G, I, J).

### Transmission electron microscopy at site 3

**Fig. 7:**
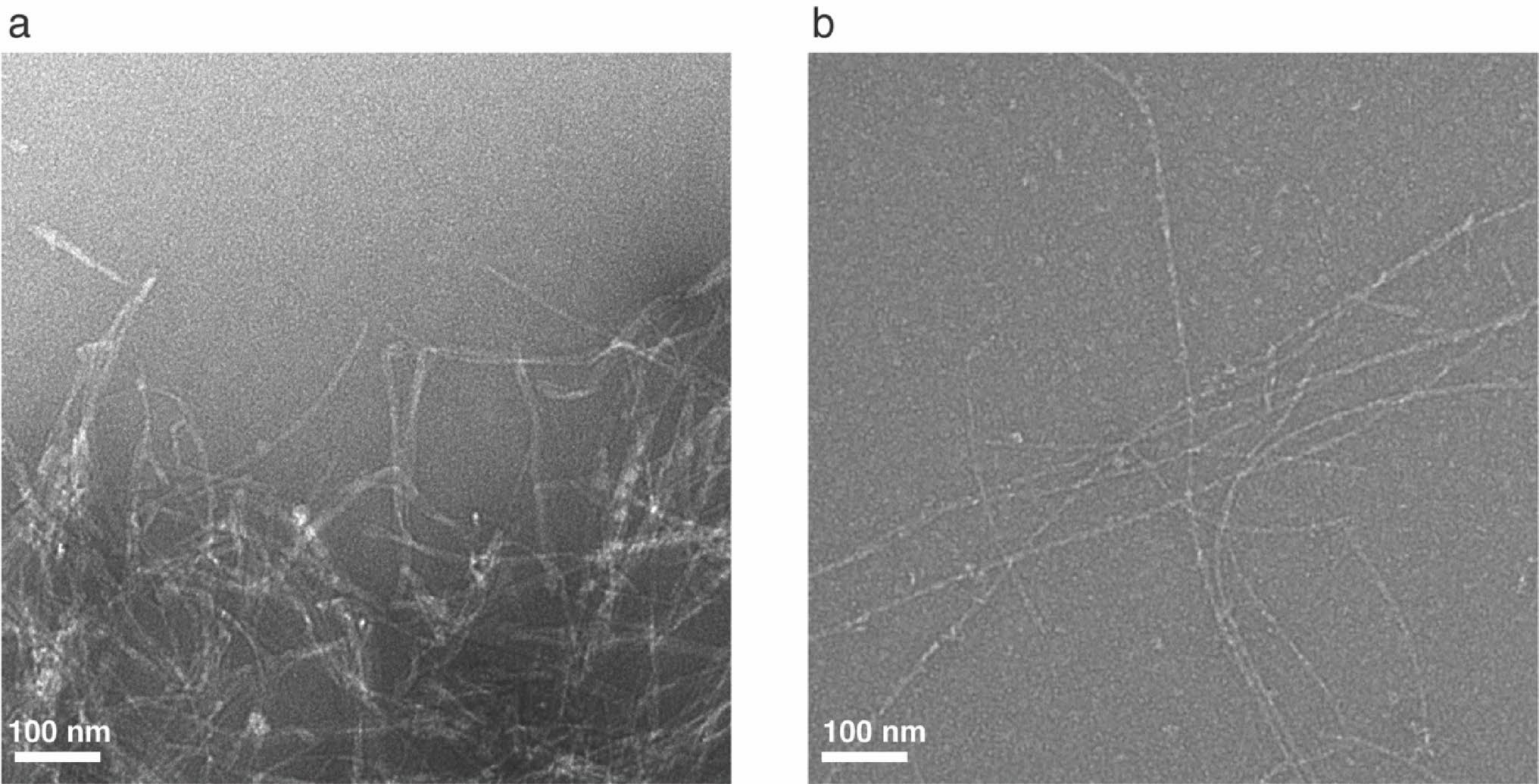
Negatively stain transmission electron microscopy (TEM) images on insulin fibrils. (**a**) TEM image representing the insulin fibrils made at Site 3 with the received insulin monomer; (**b**) TEM image showing insulin fibers aggregated at site 1. Both images have a scale bar of 100 nm and a magnification level of 45000x

At site 3, different batches of mature insulin fibrils were studied using negative stain TEM, high-speed atomic force microscopy (HS-AFM), and solid-state NMR (ssNMR). The TEM experiments were performed on two batches of fibrils: one type aggregated locally at site 3 from monomeric insulin and one aggregated at site 1. The TEM results on both samples are shown in Fig. 7a and b, respectively. As reported in previous studies (Yuzu et al. 2020; Zako et al. 2009), very long fibrils were visible in both cases.

### High-speed atomic force microscopy (HS-AFM)

**Fig. 8:**
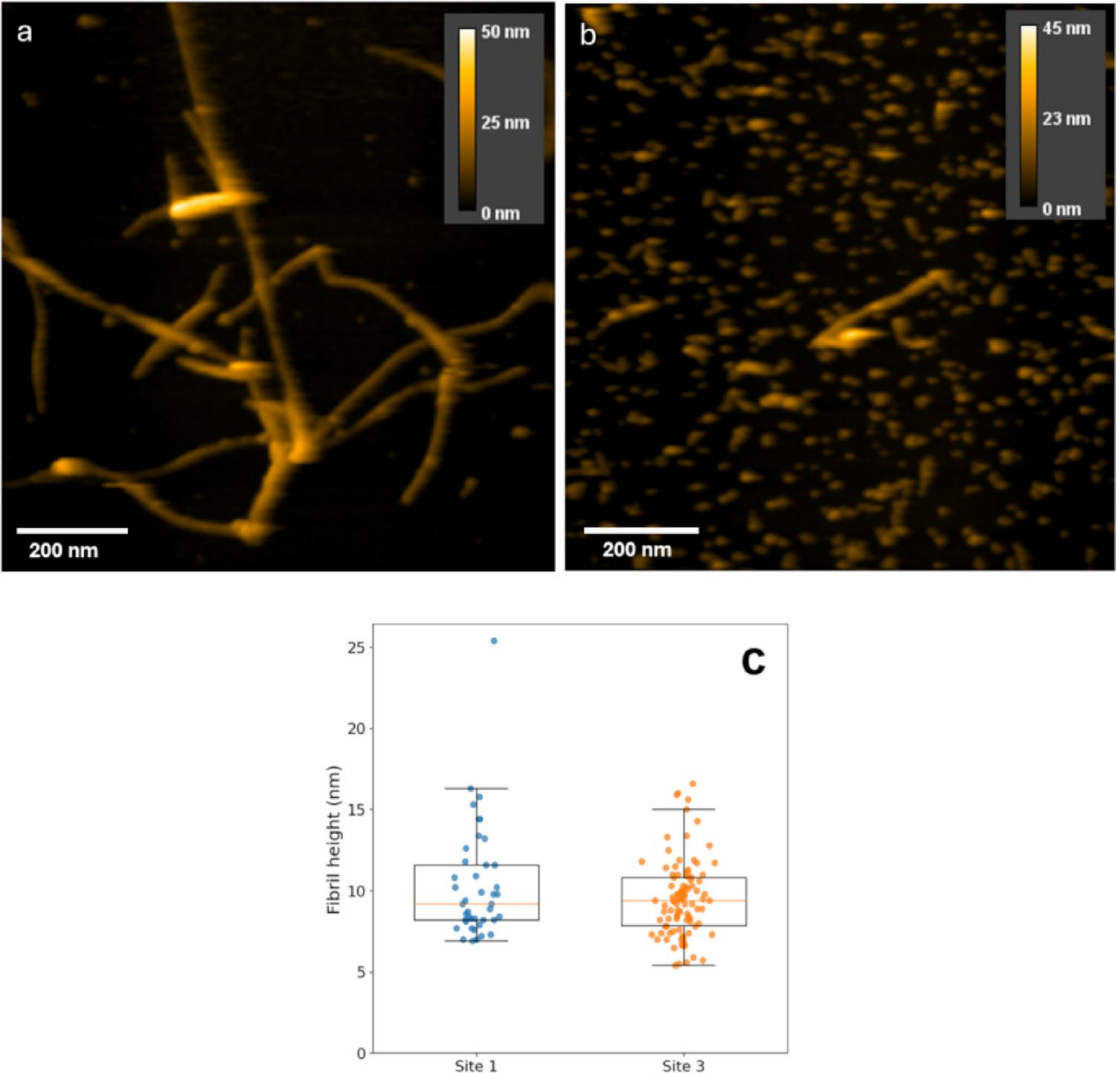
High-Speed Atomic Force Microscopy (HS-AFM) overview images on insulin fibrils. **a**) Fibrils aggregated in site 3; **b**) Fibrils aggregated in site 1. **c**) Height distribution comparison between fibrils generated at site 1 and fibrils generated at site 3

HS-AFM imaging (van Ewijk et al. 2024) of the insulin fibrils was performed at site 3. This technique has been used before to image for instance Amyloid-β and Huntingtin protein based amyloid fibrils (Watanabe-Nakayama et al. 2023; Banerjee et al. 2017; van Ewijk et al. 2025) Fig. 8 shows an overview image of the HS-AFM measurements of both groups of fibrils. In the sample prepared in site 3 (Fig. 8a), longer fibrils were numerous and smaller structures/fragments were not abundant. Meanwhile, we observed a few long fibrils and numerous smaller structures/fragments in the sample prepared in site 1 (Fig. 8b). A height distribution of fibrils generated at site 1 and site 3 respectively is shown in Fig. 8c.

**Fig. 9:**
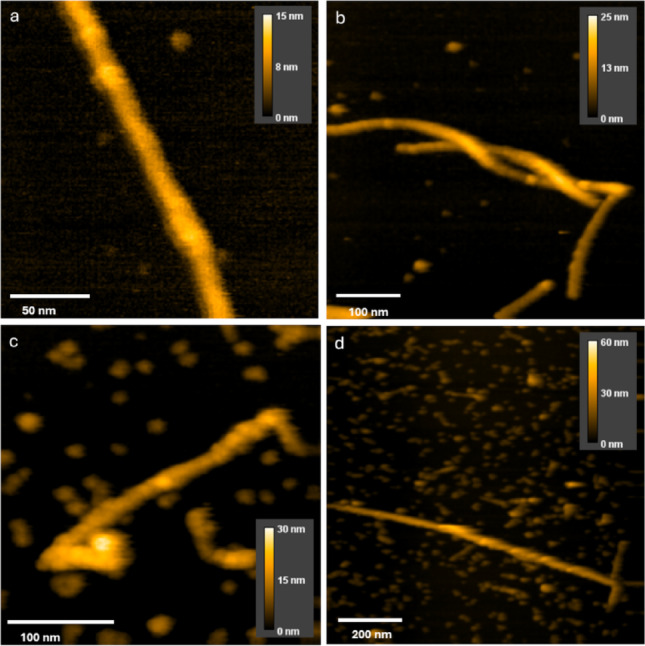
Close-up images on insulin fibrils. **a**, **b**) Fibrils aggregated in site 3; **c**, **d**) Fibrils aggregated in site 1

Figure 9 provides a closer look at individual fibrils from both groups of samples. In the case of the sample prepared in site 3 (Fig. 9a and b), the fibrils generally range between 5 and 15 nm in height, with a median of 9.4 nm (Fig. 8c). This is in agreement with the value in existing literature (Ziaunys et al. 2024; Nielsen et al. 2001) and a bit higher than AFM measurements at site 2. In Fig. 9b, we observe a case of two intertwining fibrils. Examples of fibrils prepared at site 1 are shown in Fig. 9c and d. The fibril in image 9c ranges between 12 and 16 nm in height. The fibril in image 9d constitutes a height outlier, ranging between 20 and 28 nm in height. These values are clearly higher than those observed for the fibrils prepared at site 3. However, as mentioned before, the sample prepared at site 1 shows only a few large fibrils and the majority of structures are relatively short. Those abundant, shorter structures are clearly lower than the rare fibrils, as can be appreciated in Fig. 9d. These shorter structures fit more with the expected height range.

### Solid-state NMR spectroscopy

**Fig. 10:**
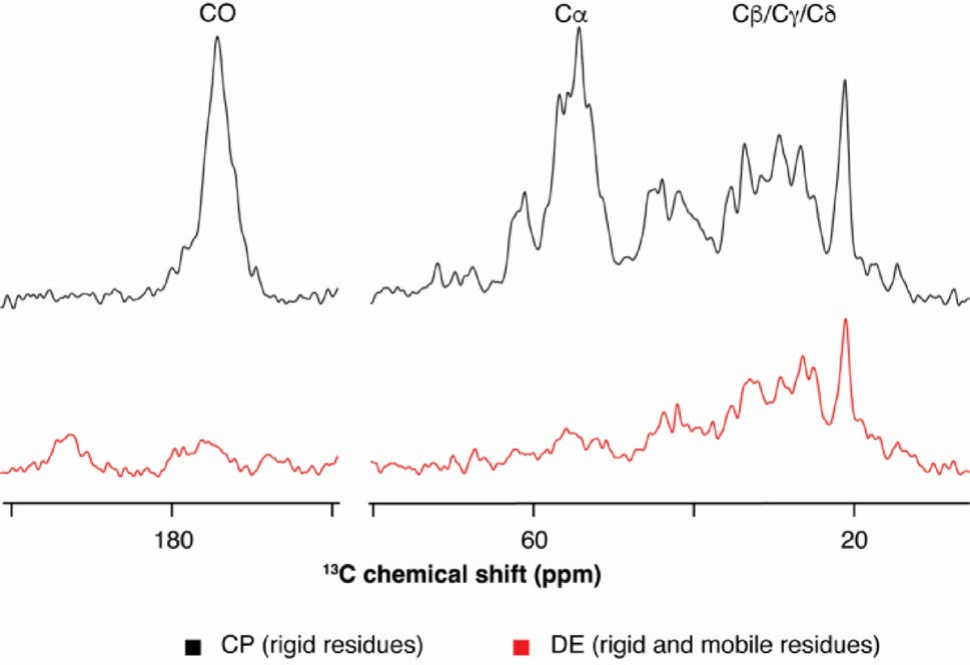
1D 13 C ssNMR experiments on unlabeled insulin fibrils. Top: CP-based ssNMR on unlabeled insulin fibrils prepared at Site 3. The aliphatic (Cα, Cβ, Cγ, and Cδ) and carboxyl (CO) regions are indicated in the CP spectrum. Bottom: 13 C direct excitation (DE) ssNMR on the same sample, show in red. The peak pattern in these spectra is consistent with prior work (see text). The relative intensities in the black and red spectra indicate that the fibrils are highly rigid

1D ^13^C magic-angle-spinning (MAS) ssNMR was conducted on unlabeled (natural abundance) insulin fibrils prepared at site 3 (Fig. 10). The lack of ^13^C enrichment limits in-depth analysis of the fibril structure, but is compatible with an overall assessment of fibril structure and dynamics (van der Wel 2018). Combined CP and DE-based experiments were used to qualitatively assess the mobility of the molecules (Matlahov and van der Wel 2018). As shown in Fig. 10, peaks in the CP spectrum are relatively narrow and defined, suggesting an ordered structure. The higher peak intensity in the CP experiment, compared to the DE experiment (red), indicates that most of the insulin residues have become rigidified in the fibril. Although the lack of labelling limited our ability to perform a detailed interpretation and assignment, we compared our findings to prior published work of relevance. A recent report (Suladze et al. 2024) described ssNMR studies of human insulin fibrils. Despite several sequence differences, we noted that these insulin fibril ssNMR spectra were qualitatively similar in the overall peak pattern, linewidths, and spectral quality. This suggested a similar structure on the molecular level. Notably, also our TEM data (Fig. 7a) revealed a similar fibril appearance to that reported for those human insulin fibrils, in terms of appearance and fibril width (approximately 5 nm).

#### SR-CD

**Fig. 11:**
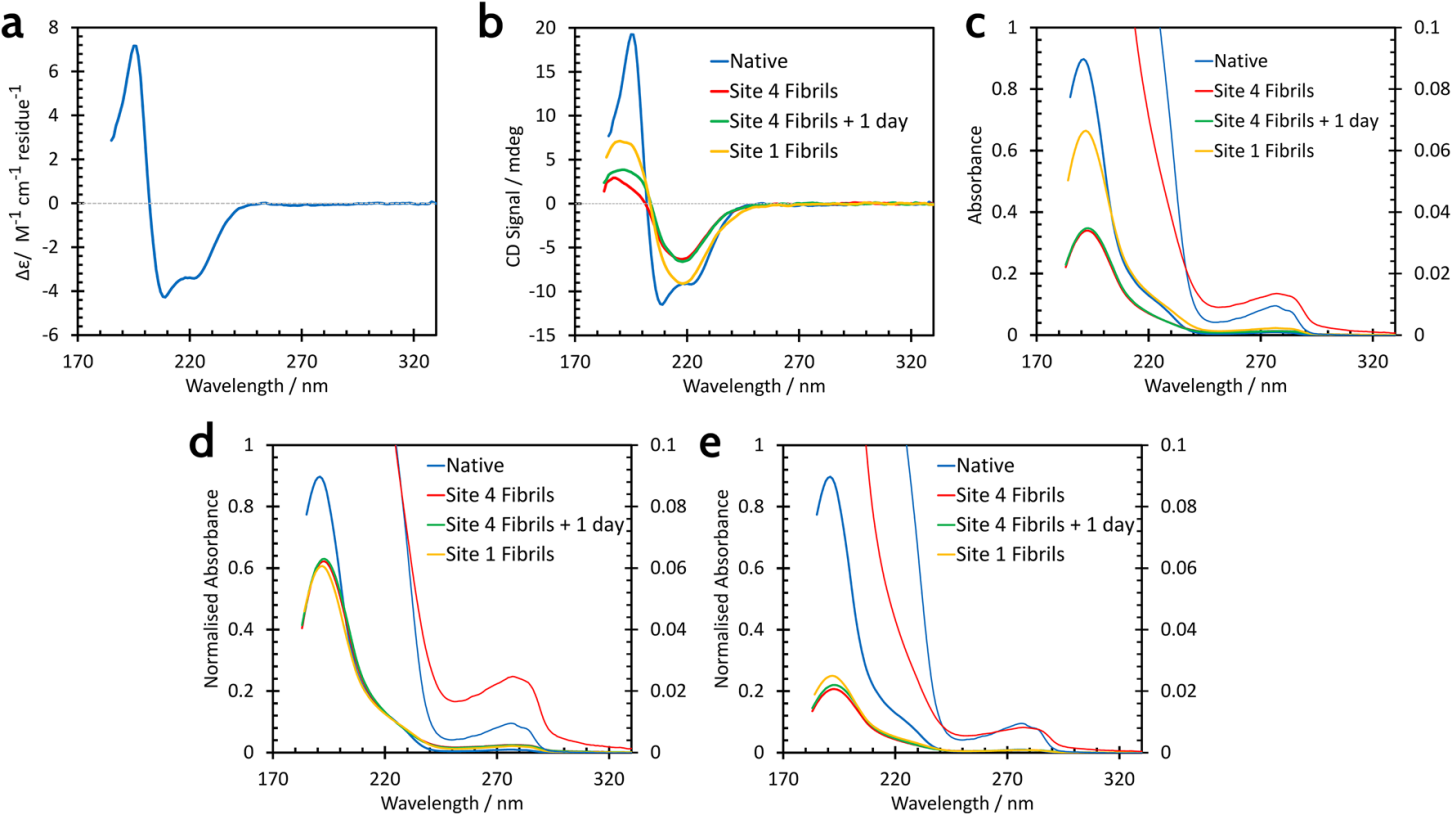
(**a**) CD spectrum of 160 µM native bovine insulin in 25 mM HCl recorded at 25 °C. (**b**) CD spectra of native and fibrillated bovine insulin samples. (**c**) Absorbance spectra of native and fibrillated bovine insulin samples. The thinner blue and red lines on the absorbance plot are the curves plotted on the right hand scale to highlight the differences in the aromatic region of the spectrum. These data are “as measured” and not normalised in any way. **d **and **e**) Normalised absorbance curves for the native and fibrillated bovine insulin samples. (**d**) normalised between 220–230 m and (**e**) between 270–290 nm. The Native and Site 4 Fibrils absorbance curves are also plotted on the expanded right scale (thin lines) to enhance the 280 nm region

CD spectra of native insulin samples were measured at a concentration of 160 µM in 25 mM HCl. Identical spectra were obtained from both a freshly prepared solution of native insulin and from the pre-prepared sample sent from site 1. Normalising the recorded CD signal (mdeg), using the pathlength of the cell and the concentration of the sample, the native spectrum is shown in Fig. 11a as Δε, in units of M^− 1^ cm^− 1^ per residue. The concentration of the sample was confirmed by the absorbance at 205 nm, Anthis and Clore (2013) which was recorded simultaneously with the CD. Analysis was carried out using SSCalcPy (Hoffmann et al. 2025) with the CDSSTRPy method and the AU-SMP180 reference set, indicates that under these conditions the native insulin sample has a considerable amount of ordered secondary structure, consisting of 46% alpha helix and 13% beta sheets, with 10% turns.

Once the sample has formed fibrils, the CD spectrum changes considerably. Figure 11b shows a comparison of the CD spectra for the native (blue curve) and fibrillated samples. The CD spectra obtained are consistent with those previously measured when investigating fibrillation of bovine insulin (Mishra et al. 2013). It is clear that the nature of the folding has changed once fibrils are formed, with the shape of the fibril CD spectra suggesting that there are now more beta-type structures. The red and green curves in Fig. 11b are for fibrils prepared at site 4 using the defined protocol with the lyophilised insulin power and 25 mM HCl buffer supplied by site 1. The CD spectrum was measured after completion of 1 day of incubation at 65 °C to allow fibrillation (red curve) and then measured again the next day with the sample having been stored at 4 °C (green curve). It is interesting that the spectrum for these fibrils had already changed after 1 day, possibly reflecting a change to return to the native form. Indeed, measurement of the CD of a sample confirmed to contain a significant amount of fibrils, prepared and sent some weeks earlier by site 1, showed that there were actually little or no fibrils and the native spectrum was largely reproduced.

The yellow curve in Fig. 11b is the spectrum of the fibrils formed after 2 days of incubation at 65 °C of the 320 µM insulin solution prepared by site 1. It was necessary to prolong the incubation time (past the 1 day included in the protocol) to allow fibrillation to occur for the “Site 1 Fibrils”, as it was clear that no fibrils had been formed after only 1 day. This was evident visually, with no obvious fibrils present in the tube and confirmed via a ThT assay. Measurement of the CD spectrum at this point also confirmed no significant fibril formation, with the spectrum recorded found to be the same as the starting native sample.

The corresponding absorbance curves of the samples, measured simultaneously with the CD, are also shown in Fig. 11c. Looking at the long wavelength region, there is a small amount of light scattering evident from the long tail of the red curve, which is unsurprising given the nature of the fibrils. However, the overall shape of the absorbance spectrum changes, highlighted when all curves are normalised either between 220 and 230 nm in the middle of the spectrum, or 270 and 290 nm, as shown in Fig. 11d and e.

Part of this difference may be due to the scattering of the fibrils, but based on the tail of the absorbance curve, this contribution appears to be too small to be responsible for the major differences seen in the lower wavelengths, plus the shape change in the 250–290 nm aromatic region.

Quantifying the amount of fibril sample to be able to compare the relative sizes of the CD signal and therefore also the type and amount of secondary structure is not straightforward. The native sample was measured at half the concentration of the prepared stock, i.e. 160 µM, in order to ensure that the absorbance was low enough to be able to measure to as low a wavelength as possible. However, the fibril samples were measured without dilution from the fibrillated 320 µM solution and the aggregates formed will likely also affect the accuracy of pipetting, thus also any estimate of concentration. The usual method of calculating the concentration of the sample via absorbance at 280 nm or our preferred 205 nm absorbance, Anthis and Clore (2013) does not appear to be an option in this case.

However, despite this limitation in the interpretation of the spectra, CD spectroscopy is an extremely useful technique to confirm and follow the formation of fibrils. Not only does the shape of the CD spectrum change with the formation of fibrils from highly ordered alpha-helix structures to being more beta-sheet like, but an obvious change in the shape of the absorbance occurs as well. The sensitivity of the CD measurements is such that a spectrum of fibrils 1 day later shows a clear change in structure, though not reflected in the absorbance spectrum, highlighting the value of CD spectroscopy in fibril studies.

#### FTIR

Fourier Transform Infrared spectroscopy (FTIR) probes molecular vibrations of molecules and for decades the technique has been used to study sample composition. More specifically, for proteins FTIR spectroscopy has been extensively used to study secondary structure, including analysis of aggregates and more recently post-translational modifications like glycosylation. The IR response of proteins and analysis of their secondary structure is well-known. Spectral shape differences in the amide I and/or amide II regions are investigated. In the case of amide I region, all different secondary structures absorb in a specific range due to different transition dipole coupling with the peptide backbone, as specified in Table 1.

**Table 1:** Assignment of amide I band based on experimental data from various research teams

Secondary structure	Band position in H_2_O (cm^-1^)	
	Center	Range
α-helix	1654	1648–1657
β-sheet	1633	1623–1641
	1684	1674–1695
Turns	1672	1662–1686
Disordered	1654	1642–1657

FTIR spectra can be obtained by different sampling methods. The simplest is the classical transmission measurements in a liquid cell. Unfortunately, water absorbs strongly in the Amide I region and therefore the pathlength should be limited to 8 μm otherwise the light will be totally absorbed by the solvent. Water can be replaced by D_2_O to avoid the strong absorption of the water and therefore the pathlength can be increased. Nowadays, the most used sampling system is attenuated total reflection (ATR). Briefly, the sample is deposited on a high refractive index crystal (like ZnSe, ZnS or diamond), the IR light goes through the crystal with an angle of 45°, the difference of refractive index between the sample and crystal is above the critical angle and therefore the light is totally reflected, but at the interface there is an evanescent wave where the light interacts with the sample. The penetration depth is usually below 1 μm, but it is sufficient to obtain IR spectra. The sample can be a liquid deposited on the crystal but the sample is often dried on the surface and measured. Measurement in solution usually requires a few µL at high concentration of protein (> 5 mg/mL) but for ATR, as the water is removed, the concentration of protein can be lower, however the amount of protein usually needs to be above 50 ng to be able to detect it.

**Fig. 12:**
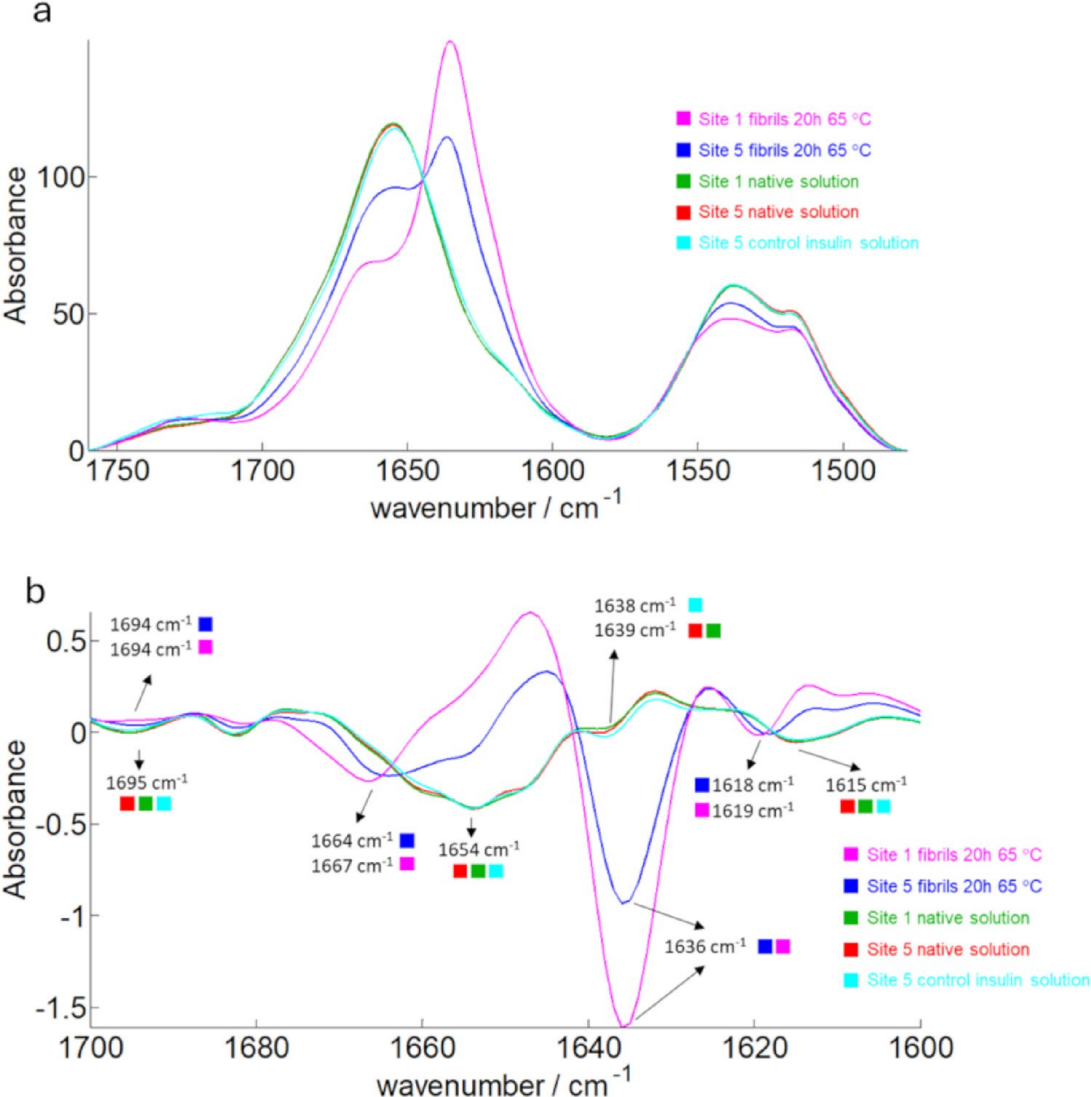
(**a**) ATR-FTIR results of the different solution of Insulin before and after fibrillation 20 h at 65 °C. Comparison of the processed spectra recorded. Only the spectral region related to proteins absorption (1760 –1478 cm^-1^) is shown. Each spectrum is identified by a unique colour indicated in the legend. (**b**) Second derivative of ATR-FTIR spectra of the different solution of Insulin before and after fibrillation 20 h at 65 °C. Comparison of the processed spectra recorded. Only the spectral region related to proteins absorption (1760 –1478 cm^-1^) is shown

The major advantages of IR spectroscopy for amyloid fibril studies are its ability to measure sample in any state (soluble or insoluble), as during the aggregation process the soluble insulin becomes insoluble amyloid fibrils, and its capability to differentiate the classic intramolecular β-sheet structure and the intermolecular β-sheet observed in the protein aggregates. ATR-FTIR spectra obtained of insulin samples before and after the aggregation are shown in Fig. 12a. The spectrum has two major bands between 1700 and 1600 cm^− 1^, corresponding to amide I and the band between 1600 and 1500 cm^− 1^ for amide II. For the native insulin sample, a major peak is observed at 1654 cm^− 1^ indicative of structure mainly due to α-helix, in comparison for the sample after aggregation the major band is at 1636 cm^− 1^ typical for β-sheet structures, and the intensity of the 1654 cm^− 1^ decreases as the α-helix structures are converted to β-sheet. To better see the structural changes Fig. 12b shows the second derivative of the spectra. The minima show the exact position of the bands.

IR spectroscopy is able to measure changes in secondary structure. There was no significant difference in the native insulin solutions prepared at sites 1 and 5, however a major difference was observed after incubation at 65 °C. Fibrillation was observed for both insulin solutions, but despite being incubated for the same amount of time, there are differences between the two IR spectra. Both show a major peak at 1636 cm^− 1^, but the intensity of this peak is different and therefore the quantity of fibrils formed during the incubation is different. It is unfortunately not possible to quantify the amount of fibrils formed, as it will require references sample with known amount of fibrils to build a calibration curve. Nevertheless, it is clear that FTIR is able to detect secondary structure variations that indicate the formation of amyloid fibrils in the insulin samples.

## Discussion and conclusions

The field of protein biophysics and the development of biophysical measurement techniques have advanced significantly, enabling the detection and monitoring of both static and dynamic properties of natively folded proteins and protein complexes. These advancements have greatly enhanced our understanding of the relationship between protein structure and function, which in turn has profoundly influenced the identification of drug targets and the development of therapeutics for diseases arising from protein misfolding. Nonetheless, methods are needed to study the dynamic process of protein misfolding and amyloid fibril formation, and the nature of various amyloid polymorphs.

### Benchmarking of sample homogeneity

With the aim to generate monomorphic amyloids from the same starting material but at different sites, we distributed bovine insulin from the same batch and bottle together with buffer of identical composition to several experimental sites. Fibrils were generated at each site according to a pre-established protocol. Surprisingly, ThT-positivity of the fibrillation reaction could not be established at all sites at the stipulated endpoint of 20 h and the incubation period was prolonged by 18–24 h for some samples whereafter ThT positivity was identified at all sites. Comparison of 4 different samples from site 1 using ThT fluorescence intensity and TEM revealed that despite identical conditions, the resulting fibrils were not homogenous in shape or in ThT fluorescence propensity (Fig. 2A-B). Even sample 1C and 1D that were run totally in parallel, as two aliquots of the same master mix, varied in ThT intensity and structural morphology deduced by TEM. In addition, when comparing sample 1 C and 1D in a 3D fluorescence scan with pFTAA, they differ in fluorescence response to this conformation sensitive amyloid dye, indicating variable structure. The obtained insulin amyloid samples at sites 2–5 were aliquoted and one aliquot from each site was returned to site 1 where ThT intensity, TEM analysis and hyperspectral fluorescence with the LCO pFTAA was performed. Again, and based on the variable results even between parallel samples at one site, a large variability of the different parameters measured was attained (Figs. 3 and 4). AFM analysis further confirmed the presence of distinct morphological characteristics among fibrils formed at different sites. Comparison of site 1, 2, and 3 samples reveals significant differences in both fibril thickness and length.

The following conclusions could be drawn from the benchmarking activities:

Bovine insulin amyloid fibril formation can rather straightforwardly be induced by subjecting the protein (1.8 mg/ml or 320 µM on a monomer basis) to fibril promoting denaturing conditions (pH 1.6 and 65 °C) for 20–48 h (Surmacz-Chwedoruk et al. 2016) While this rather harsh condition can facilitate protein hydrolysis (Mishra et al. 2007), hydrolysis or other chemical modification has not been reported for insulin but cannot be excluded. However, the fibrils that form under these conditions are quite diverse in terms of morphological ultrastructure and ThT fluorescence response (Figs. 2 and 3). This sample variability was confirmed to be attributed to amyloid fibril polymorphism by hyperspectral analysis of the conformation sensitive LCO pFTAA, which showed wide variations between samples (Figs. 4 and 5). The differences in ThT intensities and pFTAA spectral properties could potentially also be ascribed to different degrees of flocculation and sonication of the fibrils [56]. The results showed that the formed fibrils are not only variable due to differences in which sites generate the fibrils, but also fibrils formed in the same lab showed equally diverse structures. Possible ways of mitigating variability/polymorphic behaviour of insulin amyloid fibrils are:

- Extended fibril incubation times. 24 h at 65 °C predominantly used here, even up to 48 h may not be enough, although the most common lag-phase reported for acidic high temperature induced fibril formation of bovine insulin at comparable protein concentration in the literature is 4–6 h.
- Increase concentration of insulin to 1 mM and add 100 mM NaCl to increase the rate of fibril formation.
- Dialysis of dissolved lyophilized insulin to minimize the effect of variable additives from the commercial insulin (Sigma-Aldrich Cat#6634).
- Repeatability using commercial bovine insulin can be an issue due to variability originating from the natural source. Hence the use of recombinant expressed human insulin to avoid variability is advised (Sigma-Aldrich Cat# 91077 C).
- To seed fibrils with preformed fibrils to template and select a thermodynamically stable polymorph.
- Sonication of fibril samples prior to ThT or pFTAA fluorescence analysis to avoid variations in pipetting of microscopic fibril clusters rather than well dispersed fibrils.
- Analyse possible chemical modifications formed during incubation at high temperature low pH conditions, such as hydrolysis, by mass spectrometry.

### Biophysical measurement techniques utilized on amyloid samples

Even though the polymorphic nature of misfolded protein amyloid fibrils poses a challenge for standardization, it is a feature of high interest for understanding protein structure and function relationships in amyloid diseases. High resolution structure determination by cryo-EM has revolutionized the understanding of amyloid fibril structural polymorphism. More insights are also emerging during the fibril formation process by this method (Wilkinson et al. 2023; Lövestam et al. 2024). However, sample preparation, selective particle imaging of non-stacked fibrils, and image averaging of repetitive motifs can influence cryo-EM applications of certain systems. Hence, complementary biophysical methods such as those described in this work are essential to understand the process in detail. The conversion of native protein into amyloid fibrils and the features of various polymorphic structures can hence be classified using different types of biophysical methods. As summarized in this paper, fluorescence,- CD,- ssNMR,- and FTIR-spectroscopy all provide structural information of the conversion process and the presence of various fibril polymorphs. Microscopy methods TEM, HS-AFM, and AFM-SCFM provide nanoscopic information of fibril structure and morphology, where specifically AFM-SCFM and HS-AFM enable detection of fibril variations highlighting structural polymorphism on a single particle scale, even within a single fibril. Of note, potential further AFM analysis may look into structural polymorphism in the helical pitch of fibrils using a contact point reconstruction algorithm [57].
